# Diet-Induced Obesity Increases Monocyte/Macrophage Proliferation during Skin Wound Healing in Mice

**DOI:** 10.3390/cells13050401

**Published:** 2024-02-26

**Authors:** Jingbo Pang, Norifumi Urao, Timothy J. Koh

**Affiliations:** 1 Center for Wound Healing and Tissue Regeneration, Department of Kinesiology and Nutrition, University of Illinois at Chicago, 1919 W. Taylor Street, Chicago, IL 60612, USA; 2 Department of Pharmacology, State University of New York Upstate Medical University, Syracuse, NY 13210, USA

**Keywords:** obesity, wound healing, inflammation, macrophage, proliferation

## Abstract

Obesity is associated with low-grade chronic inflammation and impaired glucose metabolism, both of which are detrimental to wound healing. C-C motif chemokine receptor 2 (CCR2) plays an important role in cell recruitment during healing, and our recent studies revealed the significance of CCR2-CCL2 signaling in promoting the proliferation of pro-inflammatory monocytes/macrophages in wounds. Therefore, we sought to determine whether diet-induced obesity increases monocyte/macrophage proliferation and their accumulation in skin wounds. We first confirmed that wound closure was delayed in obese CCR2^RFP/+^ mice fed with a high-fat diet (HFD) compared to mice fed with a normal diet (ND). Using in vivo imaging and flow cytometry analysis, we found that HFD mice had significantly increased accumulation of CCR2+ monocytes/macrophages, particularly pro-inflammatory CCR2+Ly6C+ cells in wounds compared to their ND counterparts. Importantly, HFD mice exhibited an increased proliferation of wound CCR2+Ly6C+ compared to ND mice. Together, our data suggest that obesity leads to an increased proliferation and accumulation of pro-inflammatory CCR2+Ly6C+ monocytes/macrophages in skin wounds, which may contribute to delayed healing.

## 1. Introduction

Obesity is a major health issue throughout the world, affecting millions of people, increasing the risk of many chronic diseases, and incurring staggering healthcare costs [1]. Obesity and associated chronic inflammation impair the healing of various tissues and organs, including skin [2,3]. Accumulating evidence indicates that obesity can disrupt all stages of skin wound healing, including inflammation, proliferation and remodeling phases, both in humans and in animal models [2,3,4,5].

Cells from the monocyte/macrophage lineage are recognized as prominent players that orchestrate many biological processes important for wound healing, including pathogen killing, phagocytosis of debris and dead cells, promoting inflammation and its resolution, the proliferation and migration of other wound cells, and wound remodeling, all helping to restore homeostasis to the wound environment during efficient wound healing [6,7,8]. However, the persistent accumulation of pro-inflammatory monocytes/macrophages in diabetic wounds leads to chronic inflammation and impaired healing [9,10].

C-C motif chemokine receptor 2 (CCR2), is a receptor for several C-C motif monocyte chemokines, including CCL2, CCL7, and CCL8 [11]. Upon the appearance of an injury, CCR2+ monocytes quickly infiltrate the wound bed from peripheral blood, at least in part due to the aforementioned chemokines [12,13]. Previous studies have demonstrated the importance of CCR2 in normal skin wound healing; CCR2-deficient mice exhibit significantly delayed healing [12]. However, CCR2+ monocytes/macrophages have also been reported to promote inflammation and obesity-associated metabolic dysfunction in various tissues [14,15,16,17]. Although macrophage accumulation is generally thought to be due to monocyte infiltration from peripheral blood, primarily as a result of the CCL2-CCR2 axis, we recently showed that CCL2-CCR2 signaling also induces proliferation of Ly6C+ monocytes/macrophages in skin wounds [18,19]. However, whether obesity impacts such proliferation remains to be determined.

Therefore, we designed current study to determine whether proliferation contributes to the accumulation of CCR2+ monocytes/macrophages in skin wounds, using the diet-induced obese mouse model. Using in vivo imaging and flow cytometry analysis, our data indicated that obese mice had significantly delayed healing compared to lean controls, which was associated with increased accumulation of CCR2+ cells in wounds, particularly pro-inflammatory Ly6C+CCR2+ monocytes/macrophages. Importantly, obesity increased proliferation of these pro-inflammatory Ly6C+F4/80lo/-CCR2+ monocytes/macrophages, which may contribute to the delayed healing in obese mice.

## 2. Materials and Methods

### 2.1. Animals

Homozygous B6.129(Cg)-Ccr2^tm2.1Ifc^/J mice and C57Bl/6 were purchased from the Jackson Laboratory (Bar Harbor, ME, USA) and crossbred to generate heterozygous B6.129(Cg)-Ccr2^tm2.1Ifc^/J (CCR2^RFP/+^) mice in which CCR2+ cells express red fluorescent protein (RFP) to allow tracking of these cells. For the induction of diet-induced obesity, male and female CCR2^RFP/+^ mice (n = 15–17/group/sex) were fed a high-fat diet (HFD, 60 Kcal% fat, 7% Kcal/fructose, Research Diets) ad libitum for 12 weeks, beginning at 8 weeks of age; mice were thus ~20 weeks old at the time of wounding. Age-matched lean CCR2^RFP/+^ mice received a standard chow diet (ND; n = 15–17/group/sex). All mice were housed in environmentally controlled conditions with a 12 h light/dark cycle. Water and food were available ad libitum. All animal studies were approved by the Animal Care and Use Committee of the University of Illinois at Chicago.

### 2.2. Glucose Tolerance Test

At the end of the 12-week feeding period, to confirm the establishment of obesity and glucose intolerance, both HFD and ND mice (n = 8–9/group/sex) were weighed and fasted for 4 h before receiving an *i.p.* injection of 1 g/kg body weight dextrose. Blood glucose levels were measured from a tail nick immediately before the injection of glucose and at 15, 30, 60, and 90 min thereafter.

### 2.3. Wound Model

HFD and ND mice were subjected to excisional wounding of the dorsal skin with an 8 mm biopsy punch as previously described [19]. Two wounds were created on the dorsal skin of mice for flow cytometry analysis, while one wound model was used for the evaluation of CCR2+ cell accumulation. Tegaderm (3M, 1626W, St. Paul, MN, USA) was used to cover the wounds until tissue harvest. Wound closure was evaluated by digital pictures using Fiji Image J (v1.49).

### 2.4. In Vivo Imaging of CCR2+ Cell Accumulation

The accumulation of CCR2-RFP+ cells in skin wounds was measured using the Xenogen IVIS Spectrum (PerkinElmer, Waltham, MA, USA). Following wounding, mice were placed in a light-tight imaging chamber under anesthesia; fluorescence signals of CCR2-RFP were collected at as early as 2 h and up to 14 days post-wounding. The radiation efficiency of fluorescent signals was measured within the wound area throughout the healing process.

### 2.5. Flow Cytometry

Skin wounds, femoral bone marrow cells, and peripheral blood cells were collected on day 3 and 6 post-wounding for flow cytometry analysis. Skin wounds were collected using a 12 mm biopsy punch and immediately dissociated into single-cell suspension by enzymatic digestion as previously described [19]. Cells were first incubated with Zombie Violet (Biolegend, San Diego, CA, USA) to assess cell viability. Cells from bone marrow and blood were not stained with Zombie dyes due to the low percentage of dead cells (<3%). After Fc receptor blocking with anti-CD16/32 antibody (Biolegend, clone S17011E), skin cells were labeled with anti-Ly6G−BV605 (Biolegend, clone 1A8), CD11b−APC/Fire750 (Biolegend, clone CBRM1/5), F4/80−FITC (Biolegend, clone BM8), and Ly6C−Percp/cy5.5 (BD Biosciences, San Jose, CA, USA, clone AL−21) antibodies; cells from bone marrow and blood were stained with anti-Ly6G−BV421, CD11b−APC/Fire750, CD115−APC (Biolegend, clone AFS98), and Ly6C−Percp/cy5.5 antibodies.

For proliferation analysis, mice were administrated with 2 mg BrdU (Sigma-Aldrich, St. Louis, MO, USA, B9285) via *i.v.* injection through the retro-orbital vein on day 3 and day 6 post-wounding. At 4 h post-BrdU injection, mice were sacrificed for cell proliferation analysis in skin wounds, bone marrow, and peripheral blood. After surface staining of markers, cells were fixed, permeabilized, incubated with DNase-I (Sigma) using a BD Cytofix/Cytoperm™ kit, then incubated with anti-BrdU-FITC antibody (Biolegend). For cells from skin wounds, cells were incubated with FxCycle^TM^ Far Red (ThermoFisher Scientific, Grand Island, NY, USA) 30 min before acquisition, following the manufacturer’s instructions. All samples were analyzed on LSR Fortessa with HTS (BD Biosciences) cytometer. Data were analyzed using FlowJo (FlowJo LLC, Ashland, OR, USA).

### 2.6. Statistics

Data are expressed as mean ± SEM. Statistical significance of differences was evaluated by Mann–Whitney test or ANOVA. A value of *p* < 0.05 was considered statistically significant.

## 3. Results

### 3.1. High Fat Diet-Induced Obesity Results in Delayed Healing and Impaired Glucose Tolerance

**Figure 1:**
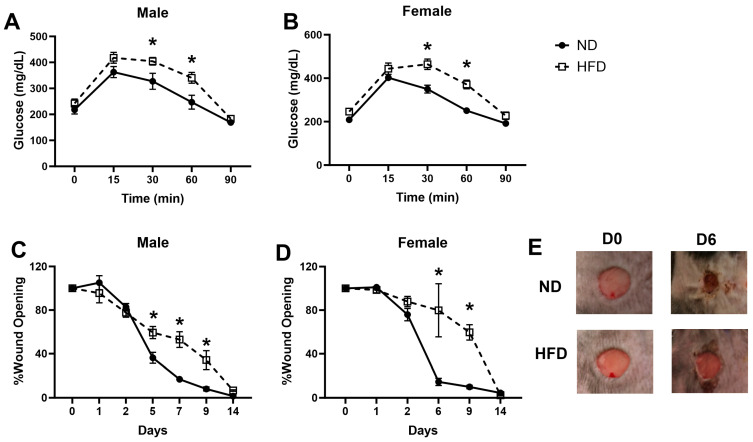
**High fat diet-induced obesity results in delayed healing and impaired glucose tolerance.** CCR2^RFP/+^ mice were fed with HFD or ND for 12 weeks to induce obesity. Glucose tolerance test was performed on 4 h fasted male ((**A**), n = 8/group) and female ((**B**), n = 9/group) mice. One 8 mm full-thickness excisional wound was created on the dorsal skin of all mice. Wound opening was calculated as the percentage of open area relative to the initial wound size on day 0 in male ((**C**), n = 5/group) and female mice ((**D**), n = 4–5/group). (**E**) Representative images of wound closure on day 0 and 6 post-wounding in male mice. Data are mean ± SEM; * *p* < 0.05 vs. ND group by two-way ANOVA.

To investigate the effects of obesity on wound healing, we fed both male and female CCR2^RFP/+^ mice with HFD for 12 weeks. As expected, both male and female HFD mice developed obesity, as indicated by a significantly higher body weight than the ND controls (male—HFD 50.1 g ± 1.5 g vs. ND 31.5 g ± 0.7 g, *p* < 0.0001; female—HFD 44.9 g ± 1.5 g vs. ND 24.6 ± 0.5 g, *p* < 0.0001). Additionally, all HFD mice showed impaired tolerance to glucose challenge compared to the ND controls (Figure 1A,B). Next, we created two full-thickness excisional wounds on the dorsal skin of the HFD and ND mice. As shown in Figure 1C–E, HFD mice showed a significantly delayed wound closure compared to the ND controls in both male and female mice, particularly at the later stage of healing. No significant differences were observed between male and female mice in glucose tolerance (Figure S1A,D) or wound closure (Figure S1B,E) in either HFD or ND mice. Together, our data confirmed glucose intolerance and impaired healing in both male and female mice fed a long-term HFD.

### 3.2. In Vivo Imaging Shows Increased CCR2+ Cell Accumulation in Skin Wounds of Obese Mice

**Figure 2:**
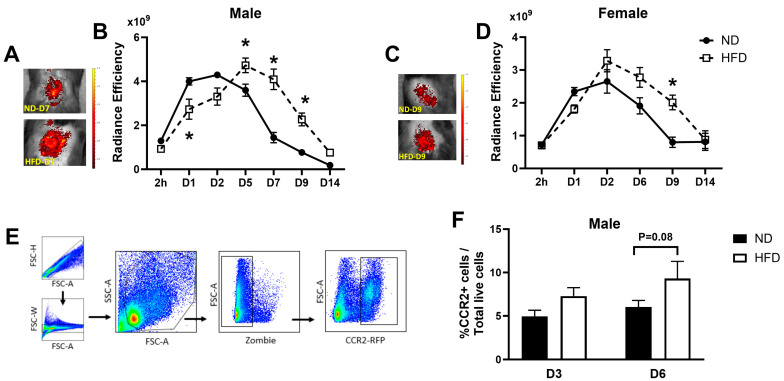
**In vivo imaging shows increased CCR2+ cell accumulation in skin wounds in obese mice.** Fluorescence signals of CCR2-RFP were collected at 2 h up to 14 days post-wounding. Representative images of CCR2-RFP signals and radiance efficiency were measured within the wound area in male ((**A**,**B**), n = 5/group) and female ((**C**,**D**), n = 4–5/group) mice. (**E**) Gating strategy for identifying Live CCR2-RFP+ cells in skin wounds in male mice. (**F**) Flow cytometry analysis confirmed a trend towards higher percentage of CCR2-RFP+ cell accumulation in HFD (open bar) than ND (black bar) mice on day 6 post-wounding (*p* = 0.08, n = 4–5/group). Data are mean ± SEM; * *p* < 0.05 vs. ND group by ANOVA.

Previous studies have indicated that CCR2 plays an important role in monocyte/macrophage recruitment and promotes inflammation during skin wound healing [12]. Therefore, we assessed the effects of obesity on the accumulation of CCR2+ monocytes/macrophages throughout the healing process. To assess CCR2+ cell accumulation in wounds, we utilized CCR2^RFP/+^ mice for which fluorescent cells within the injured area can be tracked by in vivo imaging (Figure 2A,C). In response to injury, CCR2+ cells infiltrated into the wound as early as day 1 post-wounding (Figure 2B,D). Interestingly, the accumulation of wound CCR2+ cells appeared to be delayed in male HFD mice, as the RFP signal was lower in HFD wounds than the ND counterparts on day 1 post-wounding; however, this delay was not observed in female mice (Figure 2B,D). During later stages of healing, the CCR2+ cell signal dissipated in the wounds of ND mice, but both male and female HFD mice showed a persistent accumulation of CCR2+ cells, with significantly higher RFP signals detected in wounds on day 5, 7, and 9 post-wounding compared with the ND controls (Figure 2B,D). Interestingly, the accumulation of CCR2+ cells in wounds was significantly higher in HFD male mice compared to HFD female mice at the early stages of healing, but this difference did not persist as healing progressed and was not evident in ND mice (Figure S1C,F).

We also performed flow cytometry analysis of skin wound cells isolated from male CCR2^RFP/+^ mice fed either ND or HFD to corroborate the fluorescent imaging data. On day 6 post-wounding, there was a trend towards a higher percentage of CCR2+ cells in HFD mice compared to the ND controls (*p* = 0.08, Figure 2E,F); although this difference did not reach statistical significance, the trend was consistent with our observation by IVIS imaging. Together, our data demonstrated that HFD induced a persistent elevation of CCR2+ cells associated with the impaired healing of skin wounds.

### 3.3. Obesity Leads to Increased Accumulation of CCR2+ Monocytes/Macrophages in Skin Wounds

**Figure 3:**
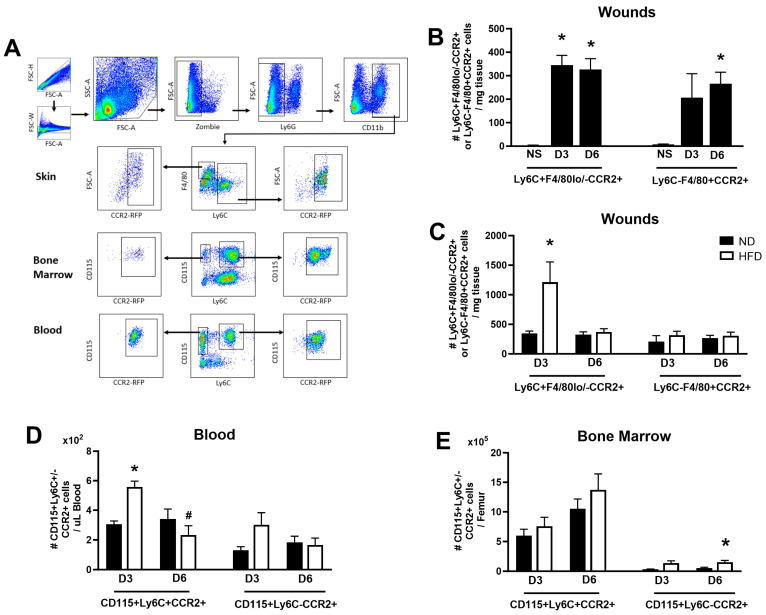
**Obesity leads to increased accumulation of CCR2+ monocytes/macrophages in skin wounds.** (**A**) Gating strategy for assessing CCR2+ macrophage/monocyte populations in skin, bone marrow, and peripheral blood. (**B**) Numbers of CCR2+Ly6C+F4/80lo/- monocytes/macrophages and CCR2+Ly6C-F4/80+ macrophages in non-injured skin (NS, n = 2) and wounds on days 3 (n = 4) and 6 (n = 6) post-injury in ND male CCR2^RFP/+^ mice. (**C**) Numbers of CCR2+Ly6C+F4/80lo/- monocytes/macrophages and CCR2+Ly6C-F4/80+ macrophages in wounds on days 3 (n = 4) and 6 (n = 6) post-injury in ND (black bar) and HFD (open bar) male CCR2^RFP/+^ mice. Numbers of CCR2+CD115+Ly6C+ and CCR2+CD115+Ly6C- monocytes in peripheral blood (**D**) and bone marrow (**E**) on day 3 and day 6 post-injury in ND and HFD male mice. Data are mean ± SEM; * *p* < 0.05 vs. ND group and ^#^ *p* < 0.05 vs. D3 group by ANOVA.

Since CCR2 can be expressed on cells other than monocytes/macrophages, we conducted flow cytometry analysis to investigate CCR2 expression specifically on two well-defined macrophage/monocyte populations that play important roles both in normal and impaired wound healing [9,18]. In response to injury, both the pro-inflammatory CCR2+ monocytes/macrophages (Live Ly6G-CD11b+F4/80lo/-Ly6C+CCR2+) and mature macrophages (Live Ly6G-CD11b+F4/80+Ly6C-CCR2+) quickly accumulated in skin wounds (Figure 3B). Consistent with genetically obese mice [18], male HFD mice exhibited a significantly higher accumulation of pro-inflammatory CCR2+Ly6C+ monocytes/macrophages in skin wounds compared to ND controls on day 3 post-wounding, while mature CCR2+F4/80+Ly6C- macrophages did not show significant differences between HFD and ND mice (Figure 3C).

Since CCR2+ monocytes are generated in bone marrow and travel through peripheral blood to reach the wound site [8], we also evaluated changes in CCR2+ monocytes after wounding in these tissues in HFD and ND mice. As shown in Figure 3D, the numbers of circulating CCR2+ monocytes (Ly6G-CD11b+CD115+Ly6C+CCR2+) were significantly higher in HFD mice than their ND counterparts on day 3 post-wounding. In contrast, the numbers of bone marrow CCR2+ monocytes (Ly6G-CD11b+CD115+Ly6C+CCR2+) were comparable between HFD and ND mice regardless of time points of wounding (Figure 3E). Moreover, the intermediate monocyte population (Ly6G-CD11b+CD115+Ly6C-CCR2+) was significantly elevated in the bone marrow of the HFD mice compared with ND controls on day 6 post-wounding (Figure 3E). In summary, these data indicated that HFD leads to the increased accumulation of pro-inflammatory CCR2+ monocytes/macrophages in wounds, which is associated with elevated circulating CCR2+Ly6C+ monocytes in blood.

### 3.4. Obesity Induces Increased Proliferation of CCR2+ Monocytes/Macrophages in Skin Wounds

**Figure 4:**
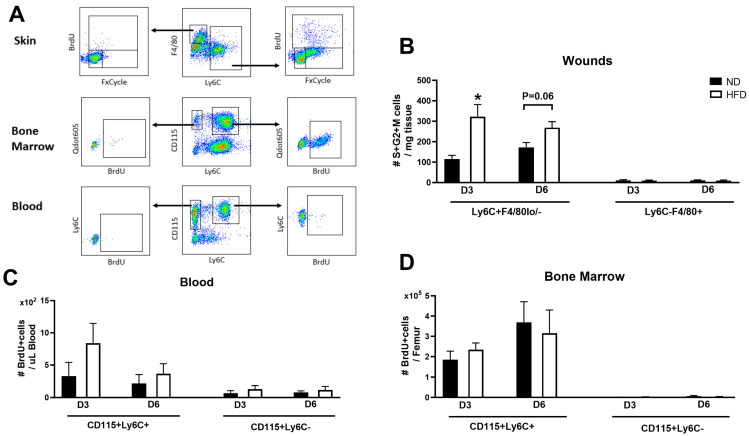
**Obesity induces increased proliferation of CCR2+ monocytes/macrophages in skin wounds.** Male mice were administered 2 mg BrdU via i.v. injection on day 3 and day 6 post-wounding. At 4 h post-BrdU injection, proliferation was evaluated. (**A**) Gating strategy for assessing proliferation of major macrophage/monocyte populations in skin, bone marrow, and peripheral blood. (**B**) Numbers of Ly6C+F4/80lo/- monocytes/macrophages and Ly6C-F4/80+ macrophages in S/G2/M phases of cell cycle (BrdU+FxCycle+) in wounds on days 3 and 6 post-injury in ND (black bar) and HFD (open bar) mice. (**C**,**D**) Numbers of CD115+Ly6C+ and CD115+Ly6C-monocytes in S phase of cell cycle (BrdU+) in peripheral blood and bone marrow, respectively. n = 4/group; Data are mean ± SEM; * *p* < 0.05 vs. ND group by ANOVA.

In addition to the infiltration of blood monocytes, our previous studies demonstrated an important contribution of monocyte/macrophage proliferation to their accumulation in the skin wounds of non-obese mice, which is enhanced in genetically obese and diabetic mice [18,19]. Thus, we sought to determine whether monocyte/macrophage proliferation was also increased in the wounds of HFD mice. Unfortunately, the CCR2-RFP signal was lost after the cell fixation and permeabilization process needed for assessing proliferation. Therefore, we were unable to use this marker in our proliferation assay. All mice were administered BrdU, and cell proliferation was evaluated by flow cytometry analysis of BrdU and cell cycle dye labeling at 4 h post-injection (Figure 4A). Consistent with our previous findings in genetically obese mice, HFD mice showed significantly higher numbers of pro-inflammatory monocytes/macrophages at the S/G2/M phases of the cell cycle on day 3 post-wounding compared to the ND controls, with a similar trend towards higher proliferation on day 6 (*p* = 0.06). In contrast, mature macrophages maintained a low proliferation rate in the skin wounds of both HFD and ND mice (Figure 4B). In blood and bone marrow, the numbers of cells incorporating BrdU were not significantly different between HFD and ND mice at any day post-wounding (Figure 4C,D). In short, our data indicate that HFD increases the proliferation of pro-inflammatory CCR2+ monocytes/macrophages in skin wounds but not in peripheral blood nor in bone marrow.

## 4. Discussion

Obesity leads to persistent accumulation of monocytes/macrophages and chronic inflammation in skin wounds, contributing to impaired healing [3,5]. In this study, we utilized in vivo imaging and flow cytometry analysis to track the accumulation and proliferation of CCR2+ monocytes/macrophages in skin wounds. Our data demonstrated increased proliferation of pro-inflammatory Ly6C+CCR2+ monocytes/macrophages in wounds of HFD mice compared to ND controls, contributing to their persistent accumulation as well as delayed healing.

Obesity leads to chronic low-grade inflammation and dysregulated glucose metabolism, both of which are detrimental to wound healing in humans and rodents [2,3,20]. Our group and others have previously demonstrated sustained inflammation, including persistent accumulation of inflammatory monocytes/macrophages and impaired healing of skin wounds in genetically obese and diabetic *db/db* mice [18,21,22]. Mice fed a HFD, which is considered a model of obesity and insulin resistance or pre-diabetes, have also been reported to be associated with delayed healing [4,5]. In our study, we confirmed delayed healing in HFD mice associated with glucose intolerance. Using in vivo imaging andCCR2^RFP/+^ mice, we demonstrated increased accumulation CCR2+ cells in wounds of HFD mice compared to ND mice during the mid-proliferative to late stages of healing. Moreover, flow cytometry analysis confirmed the in vivo imaging data, demonstrating that obesity increased accumulation of pro-inflammatory CCR2+monocytes/macrophages (CCR2+Ly6C+) but not mature macrophages (CCR2+Ly6C-F4/80+) in wounds. These data indicate that obesity may influence healing via a persistent inflammatory response, resulting from sustained accumulation of pro-inflammatory monocytes/macrophages.

CCR2 plays important roles in cell chemotaxis and promoting the inflammatory response during wound healing in mice [12]. Along with increased Ly6C+CCR2+ cells in skin wounds, we also observed elevated levels of circulating CCR2+CD115+Ly6C+ in the peripheral blood of HFD mice compared with ND controls on day 3 post-injury, suggesting that obesity increases production and/or mobilization of these cells in bone marrow. Indeed, previous studies have shown that obesity can modify the production and epigenetics of myeloid progenitor cells in bone marrow and in turn leads to leukocytosis and inflammation in peripheral tissues [23,24]. In addition, our recent studies demonstrated that wound Ly6C+ monocytes/macrophages proliferate locally, which contributes to their accumulation in wounds [19], while diabetes leads to significantly elevated proliferation of Ly6C+ monocytes/macrophages, contributing to their persistent accumulation in these mice [18]. Importantly, this process is likely driven by the CCL2-CCR2 signaling pathway that is enhanced in diabetic mice [18]. Interestingly, in the diet-induced obese model, we also observed significantly more CCR2+Ly6C+ cells at S/G2/M stages of the cell cycle in wounds in HFD than ND mice on day 3 post-injury, while this number was comparable on day 6, suggesting that the increased accumulation of CCR2+Ly6C+ monocytes/macrophages may result from increased proliferation in wounds as well as increased infiltration from blood.

Although it is possible that monocytes could incorporate BrdU in bone marrow and quickly emigrate to the wound bed, we did not detect significant differences in BrdU+ monocytes between HFD vs. ND mice in either bone marrow or peripheral blood, suggesting that Ly6C+ monocytes/macrophages proliferate in wounds. Moreover, differences in the impact of HFD on monocyte proliferation between bone marrow, blood, and wounds indicated that the wound environment, instead of cell-intrinsic factors, is key for inducing monocyte/macrophage proliferation in skin wounds. Indeed, previous studies have highlighted environmental factors which may affect macrophage proliferation in tissues, including IL-1β, IL-6, IL-4, and M-CSF [25,26,27,28]. Nonetheless, obesity likely impacts many different cells and pathways, and we cannot exclude contributions to monocyte/macrophage accumulation in wounds and impaired healing by other factors in HFD versus ND mice, and the mechanisms involved requires further study. Such mechanisms could involve altered monopoiesis in bone marrow or altered trafficking to wounds, as well as proliferation in wounds [24,29,30,31]. Finally, we note that the time point of peak proliferation differed between diet-induced obese versus genetic diabetes models, which could be due to the difference in severity of diabetes and chronic inflammation in these models [5,32]. Whether CCL2-CCR2 signaling promotes monocyte/macrophage proliferation in wounds of HFD mice as it does in diabetic *db/db* mice remains to be determined.

We note that previous studies reported that initial expansion of the adipose tissue macrophage pool in early-stage HFD-induced obesity in mice was due primarily to the proliferation of resident cells [33,34]. During the progression of obesity with continued HFD, both infiltration and proliferation contributed to further adipose tissue macrophage expansion. We also observed an early increase in the proliferation of wound monocytes/macrophages in HFD mice, consistent with the adipose tissue studies. Whether there is crosstalk between subcutaneous adipose tissue macrophages and wound macrophages is an intriguing question and awaits further study [35,36,37].

Finally, sex differences have been reported in previous studies of obesity-associated complications, as female mice were protected from HFD-induced metabolic dysfunction [38,39]. However, the influence of sex on skin wound healing remains unclear [4,40,41]. In the present study, diet-matched male and female CCR2^RFP/+^ mice showed no differences in glucose tolerance or wound closure. CCR2+ cell accumulation was increased in wounds of HFD male mice compared to HFD female mice at early stages of healing; however, no significant differences were observed between sexes at later stages of healing. In the end, the altered early CCR2+ cell accumulation between sexes did not appear to influence the kinetics of wound closure.

In summary, our study demonstrated that diet-induced obesity and pre-diabetes leads to the increased proliferation of pro-inflammatory CCR2+Ly6C+ monocytes/macrophages in skin wounds, contributing to their enhanced accumulation of in these wounds. Future studies should explore the underlying mechanisms of this obesity-associated increase in monocyte/macrophage behaviors and the inhibition of such changes to improve healing.

## Data Availability

Dataset available on request from the authors.

## Supplementary Materials

The following supporting information can be downloaded at: https://www.mdpi.com/article/10.3390/cells13050401/s1, Figure S1: Comparisions of male and female CCR2RFP+ mice fed with HFD or ND for glucose tolerance, wound closure and CCR2+ cell accumulation.
