# Supplementary figures and images for: Diet-Induced Obesity Increases Monocyte/Macrophage Proliferation during Skin Wound Healing in Mice
Source: Cells. 2024 Feb 26;13(5):401. doi: 10.3390/cells13050401 (PMC10930651; doi:10.3390/cells13050401)

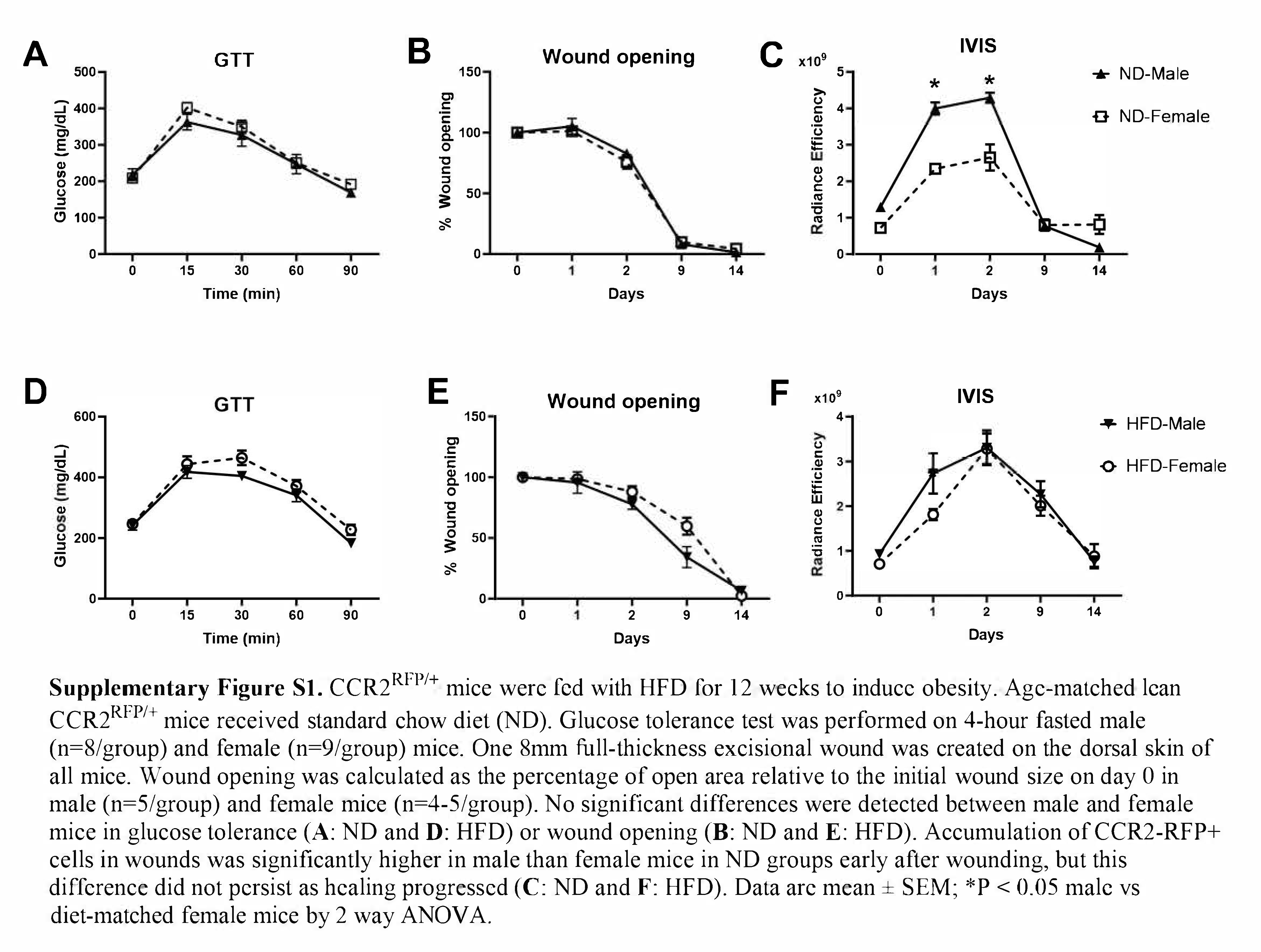

Supplement: Supplementary file 1 [file cells-13-00401-s001.zip › cells-2857267-supplementary.tif]
